# Increased pelvic incidence may lead to arthritis and sagittal orientation of the facet joints at the lower lumbar spine

**DOI:** 10.1186/1471-2342-13-34

**Published:** 2013-11-05

**Authors:** Thorsten Jentzsch, James Geiger, Samy Bouaicha, Ksenija Slankamenac, Thi Dan Linh Nguyen-Kim, Clément ML Werner

**Affiliations:** 1 Division of Trauma Surgery, Department of Surgery, University Hospital Zuerich, Zuerich, Switzerland; 2 Institute of Diagnostic and Interventional Radiology, University Hospital Zuerich, Zuerich, Switzerland

**Keywords:** Pelvic incidence, Age, Gender, Lumbar Lordosis, Facet joint arthritis, Orientation

## Abstract

**Background:**

Correct sagittal alignment with a balanced pelvis and spine is crucial in the management of spinal disorders. The pelvic incidence (PI) describes the sagittal pelvic alignment and is position-independent. It has barely been investigated on CT scans. Furthermore, no studies have focused on the association between PI and facet joint (FJ) arthritis and orientation. Therefore, our goal was to clarify the remaining issues about PI in regard to (1) physiologic values, (2) age, (3) gender, (4) lumbar lordosis (LL) and (5) FJ arthritis and orientation using CT scans.

**Methods:**

We retrospectively analyzed CT scans of 620 individuals, with a mean age of 43 years, who presented to our traumatology department and underwent a whole body CT scan, between 2008 and 2010. The PI was determined on sagittal CT planes of the pelvis by measuring the angle between the hip axis to an orthogonal line originating at the center of the superior end plate axis of the first sacral vertebra. We also evaluated LL, FJ arthritis and orientation of the lumbar spine.

**Results:**

596 individuals yielded results for (1) PI with a mean of 50.8°. There was no significant difference for PI and (2) age, nor (3) gender. PI was significantly and linearly correlated with (4) LL (p = < 0.0001). Interestingly, PI and (5) FJ arthritis displayed a significant and linear correlation (p = 0.0062) with a cut-off point at 50°. An increased PI was also significantly associated with more sagitally oriented FJs at L5/S1 (p = 0.01).

**Conclusion:**

PI is not correlated with age nor gender. However, this is the first report showing that PI is significantly and linearly associated with LL, FJ arthritis and more sagittal FJ orientation at the lower lumbar spine. This may be caused by a higher contact force on the lower lumbar FJs by an increased PI. Once symptomatic or in the event of spinal trauma, patients with increased PI and LL could benefit from corrective surgery and spondylodesis.

## Background

Pelvic rotation has emerged from the genesis of an erect position of the human spine [1]. Nowadays, a proper sagittally oriented pelvis, which acts as a basis for the building block of the entire spine, and an ideal lordotic curvature of the spine equilibrate each other in regard to overall spinal balance [2,3]. Nevertheless, aging and spinal deformities, such as spondylolisthesis may change spinal balance [4]. Thus, the establishment of a neutral upright sagittal alignment with the pelvis and spine in sync is essential in the management of spinal disorders [5,6].

**Figure 1:**
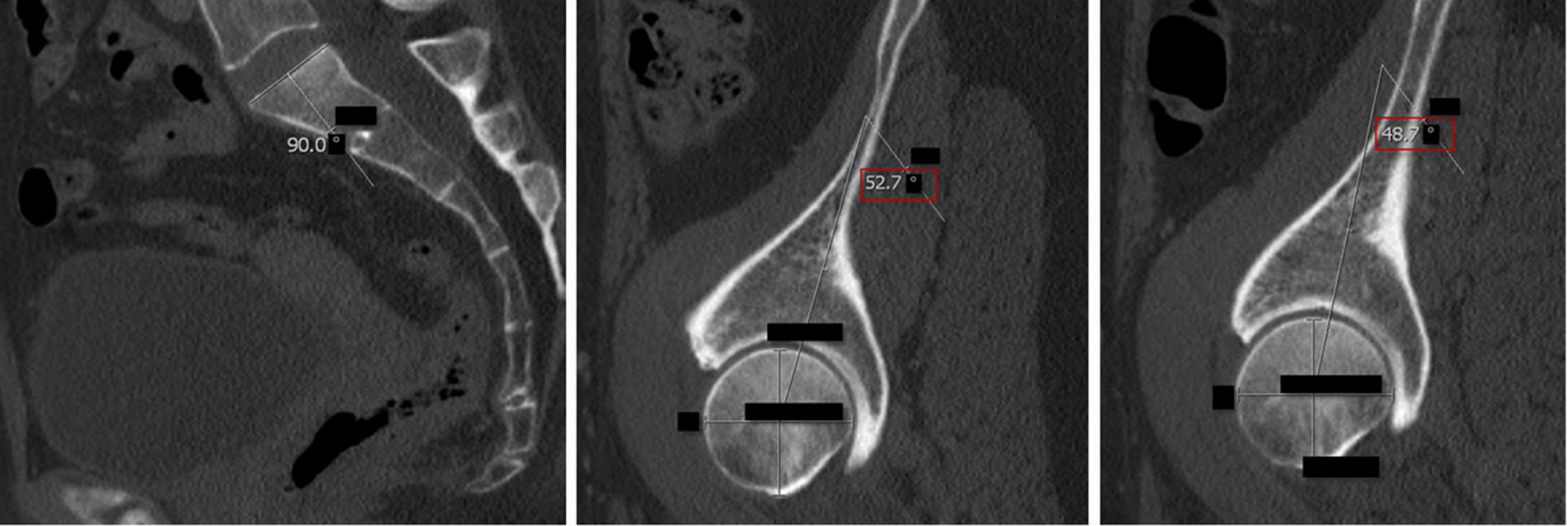
**Pelvic Incidence (PI): The PI was determined on sagittal CT planes of the pelvis.** A line was drawn along the axis of the superior end plate of S1 (left image). Then, originating at the center of this axis, an orthogonal line was drawn (left image). Secondly, the middle of the femoral head was determined by the intersecting point of a vertical and horizontal line within the femoral head (middle and right image). Finally, a line was drawn from the middle of the each femoral head to the center of the superior end plate axis and the angle was measured in regard to the orthogonal line originating at this point (middle and right image). The red box indicates the PI. The blacked out numbers were disregarded because they were created automatically by our software and contained irrelevant information. In order to acquire the superposition of the two femoral heads, left and right, the PI was measured for both sides and the mean was stated.

Although other parameters have been suggested to be superior in the study of spinal balance, pelvic incidence (PI) remains the most studied parameter [7]. It was introduced by Duval-Beaupère et al. [8] in 1992. Describing the sagittal pelvic alignment, it constitues a true anatomic parameter, since it does not change with position, e.g. standing or supine [9]. This attributes to the fact that the sacrum does not move within the rigid pelvic ring, but rotates around the bicoxofemoral axis as a whole unit [10,11]. Measurements are carried out by putting the center of the superior end plate of the first sacral vertebra in relation to the bicoxofemoral axis (Figure 1). Normal values range around 57°, with a variability of up to 10°, whereby higher values indicate a more tilted pelvis [12-15]. Nonetheless, the optimal spinal balance remains poorly definced [16].

Yet, there are a handful of remaining issues about the PI. (1) It has been studied extensively on X-rays [17,18]. But overlap or magnification of structures my falsify the measured angle [7,17]. Furthermore, very few studies [4,12,13] in the English literature have investigated the PI on CT scans, which are more more precise and more commonly used nowadays. (2) PI has been reported to increase until the age of ten and than stabilize [19-21], but other reports [22-26] have shown an increase later on during life, especially with spinal deformities, such as spondylolisthesis, or sacral fractures. (3) Even though most studies [14,27-29] have not found a gender difference, another study [18] has documented significant higher values for females. (4) Interestingly, PI may increase in order to compensate for a decrease in lumbar lordosis (LL) [24,25]. A simple predictive equation has been proposed recently [30]: LL = PI +9° (+/− 9°). (5) Facet joint (FJ) arthritis may arise from several misbalanced forces, such as increased LL, which leads to higher contact forces on the FJs, compression, rotation, and shear as well as more sagittal orientation of the lower lumbar spine, which may lead to spondylolysis and spondylolisthesis [31-36]. However, this may be prevented by compensatory mechanisms of the pelvis and a previous osteologic study [37] has linked increased pelvic lordosis to FJ arthritis at L5/S1. Other previous studies [38,39] have also found an association between the pelvic geometry and lumbar degenerative processes. However, none have focused on the association between PI and FJ arthritis, let alone PI and FJ orientation. Therefore, we hypothesized that increased PI is associated with FJ arthritis and changes in FJ orientation.

Therefore, our goal was to clarify the remaining issues about PI in regard to (1) physiologic values, (2) age, (3) gender, (4) LL and, according to our main hypothesis (5) FJ arthritis and orientation using CT scans.

## Methods

The study has been approved by the local research ethics review committee (Kantonale Ethikkommission Zürich (KEK-ZH)-Nr.2011-0507). We included and retrospectively analyzed CT scans of 620 individuals (2480 functional units consisting of two FJs and one intervertebral disc on each level between L2 and S1) [40], with a median age of 39 (IQR 27–54), who presented to our traumatology department and underwent a whole body CT scan, including the pelvis and lumbar spine, between 2008 and 2010. Exclusion criteria involved fractures of the lumbar spine and pelvis that may have changed the spino-pelvic alignment and CT studies that did not include sagittally reconstructed pelvic cross-sections. A dual-source computed tomography scanner (Somatom Definition, Siemens Healthcare, Forchheim, Germany) was used [41]. Our study utilized CT scans instead of plain radiographs, because there is a paucity on studies about the PI and FJs are more accurately displayed [12,13,42].

**Figure 2:**
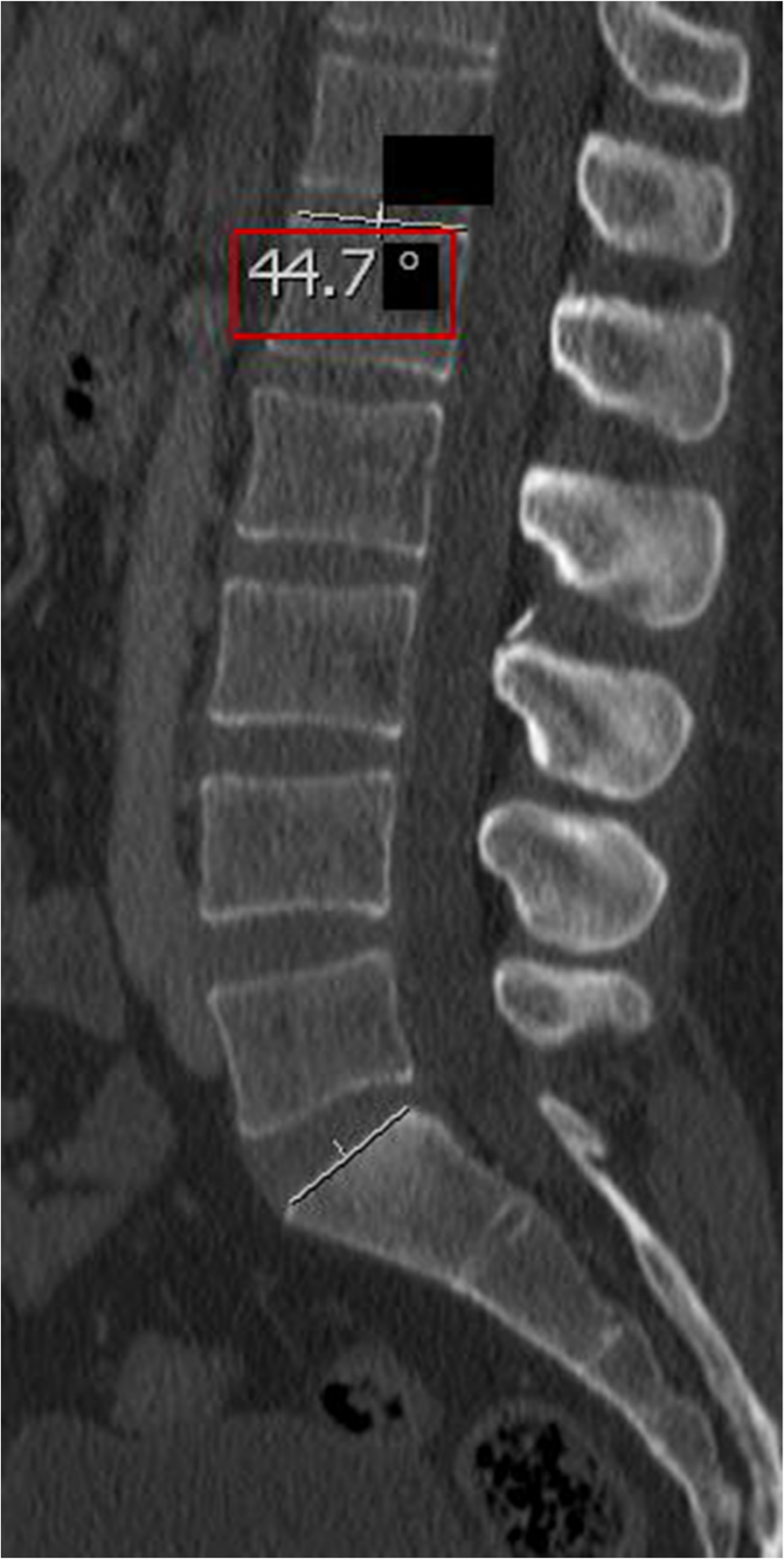
**Lumbar Lordosis (LL): LL was evaluated on median sagittal slides by measuring the angle between the superior endplates of L1 and S1, based on the definition of Stokes and the Scoliosis Research Society [**[27]**,**[44]**].** The red box indicates the PI. The blacked out numbers were disregarded because they were created automatically by our software and contained irrelevant information.

**Figure 3:**
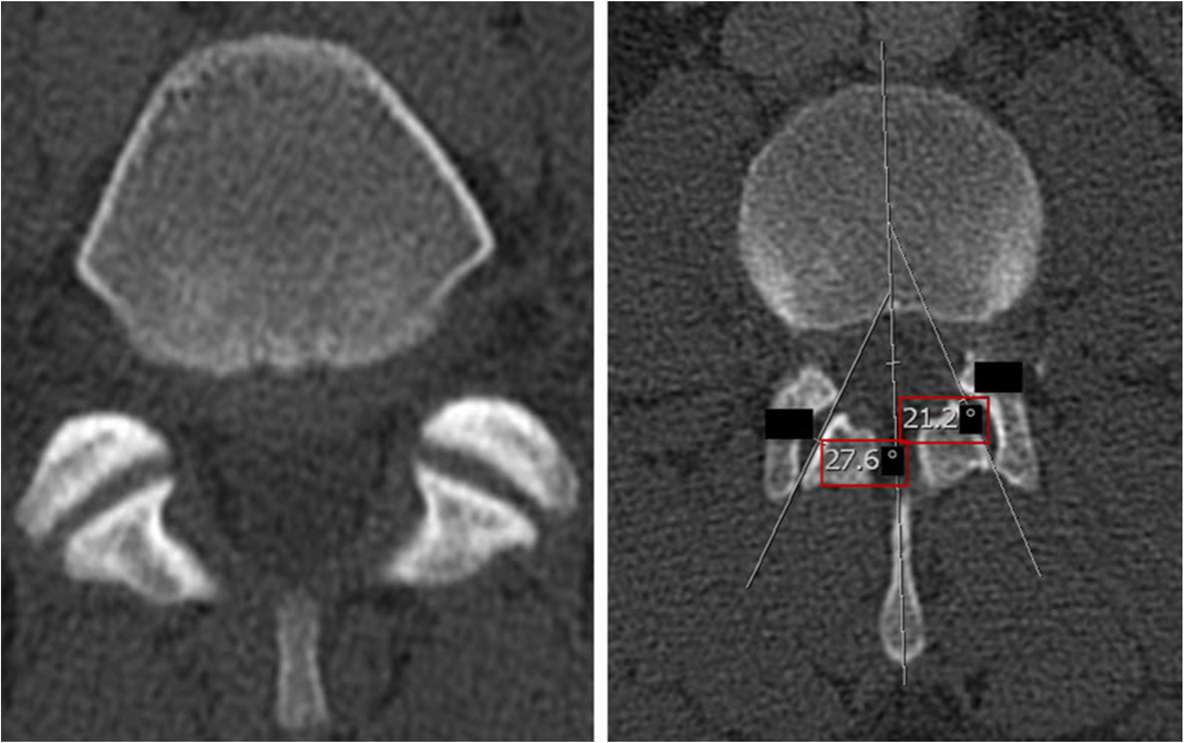
**Facet Joints (FJs): FJ orientation was evaluated by measuring the angle between the midline of the sagittal plane and the midline of the FJ as described by Schuller and Mahato [**[49]**,**[50]**].** Coronal FJ orientation is shown on the left side, whereas sagittal orientation including measurement of FJ orientation is shown on the right side. The red box indicates the PI. The blacked out numbers were disregarded because they were created automatically by our software and contained irrelevant information.

(1) The PI was determined on sagittal CT planes of the pelvis using the AGFA® Impax viewer by measuring the angle between the hip axis to an orthogonal line originating at the center of the superior end plate axis of the first sacral vertebra [8] (Figure 1). Precisely, this was done in the following manner: Firstly, a line was drawn along the axis of the superior end plate of S1. Then, an orthogonal line originating at the center of this axis was drawn. Secondly, the middle of the femoral head was determined by the intersecting point of a vertical and horizontal line within the femoral head. Finally, a line was drawn from the middle of the each femoral head to the center of the superior end plate axis and the angle was measured in regard to the orthogonal line originating at this point, In order to acquire the superposition of the two femoral heads, left and right, the PI was measured for both sides and the mean was stated. (2) Individuals were grouped into different age groups according to low, i.e. 40 years, and high, i.e. 70 years, cut-off points chosen by Kalichman et al. [43] as well as the assumption of different activity levels and degenerative processes in younger individuals ≤ 30 years, middle-aged individuals between 31–50 years and aging individuals between 51–70 years. The first group included individuals ≤ 40 and ≥ 41 years and the second group included individuals ≤ 30 years, 31–50 years, 51–70 years and ≥ 71 years. (3) Gender was also evaluated. (4) LL was evaluated on the middle of the sagittal planeby measuring the angle between the superior endplates of L1 and S1, based on the definition of Stokes and the Scoliosis Research Society [27,44] (Figure 2). The middle of the sagittal plane could be easily determined in Agfa® Impax viewer by a coexisting alignment line at the axial plane that can be viewed on the frame right next to the sagittal plane. (5) FJs of the lumbar spine were evaluated between the second lumbar and the first sacral level [45] (Figure 3). Axial planes with the largest intersecting set of the superior and inferior FJ process were chosen. Assessment of FJ arthritis was carried out as previously described in similar studies, where a grading scale described by Pathria was used [46,47]. Grade 0 (normal) indicates a normal facet joint, whereas grades 1 – 3 display increasing signs of FJ arthritis with each grade including signs of the lower grade. Grade 1 (mild) shows joint space narrowing, grade 2 (moderate) demonstrates sclerosis and grade 3 (severe) reveals osteophytes [48]. FJ orientation in the axial plane was evaluated by measuring the angle between the midline of the sagittal plane and the midline of the FJ as described by Schuller and Mahato [49,50]. The midline of the sagittal planes corresponds to a line drawn through the center of the vertebral body and spinous process. Therefore, each FJ was compared against this line. The overall FJ orientation was calculated by averaging the angles between the right and left side of the FJs. We used absolute angles, indicating that we did not consider rotation in one direction as positive nor rotation in the opposite direction as negative. The FJ orientation was labeled as coronal if angles were > 45° and sagittal if angles were ≤ 45° [51].

All statistical analysis was performed by the Institute for Social and Preventive Medicine, Division of Biostatistics at the University of Zuerich, using the R program [52]. In a first step of the analysis, we expressed distribution of variables using means and standard deviation (SD) for normally distributed data, and medians and interquartile ranges for non-normally distributed data. We tested data for normality with the Kolmogorow-Smirnow test and performed quantile-quantile plots of dependent variables. Several different statistical approaches were applied to test the remaining issues mentioned above and our main hypothesis [53], which assumed an association between an increased PI and FJ arthritis as well as changes in FJ orientation. (1) PI is a numerical measure without normal distribution, therefore simple linear regression models were applied. Therefore, PI was log transformed. An F-Test was used for nominal explanatory variables, such as (2) age, (3) gender and (5) FJ orientation. To compare PI with the numerical measure (4) LL, a linear regression was used. (5) FJ arthritis is an ordinal measure and in order to compare it to PI, which was not log transformed, FJ arthritis was used as an outcome and a proportional odds model was performed. This study is an observational study, which means that analysis follows a descriptive and exploratory form and p-values are interpreted as a quantitative measure of the evidence against the null hypothesis. Significant difference was assumed if p < 0.05.

## Results

1) PI

Of our 620 individuals, who underwent a whole-body CT scan, 596 individuals yielded results for PI. 24 (3.9%) individuals could not be evaluated because the pelvis had not been imaged or reconstructed sagittally. The median for PI was 49.9° (IQR 43.2°-57.7°).

2) PI and Age

**Figure 4:**
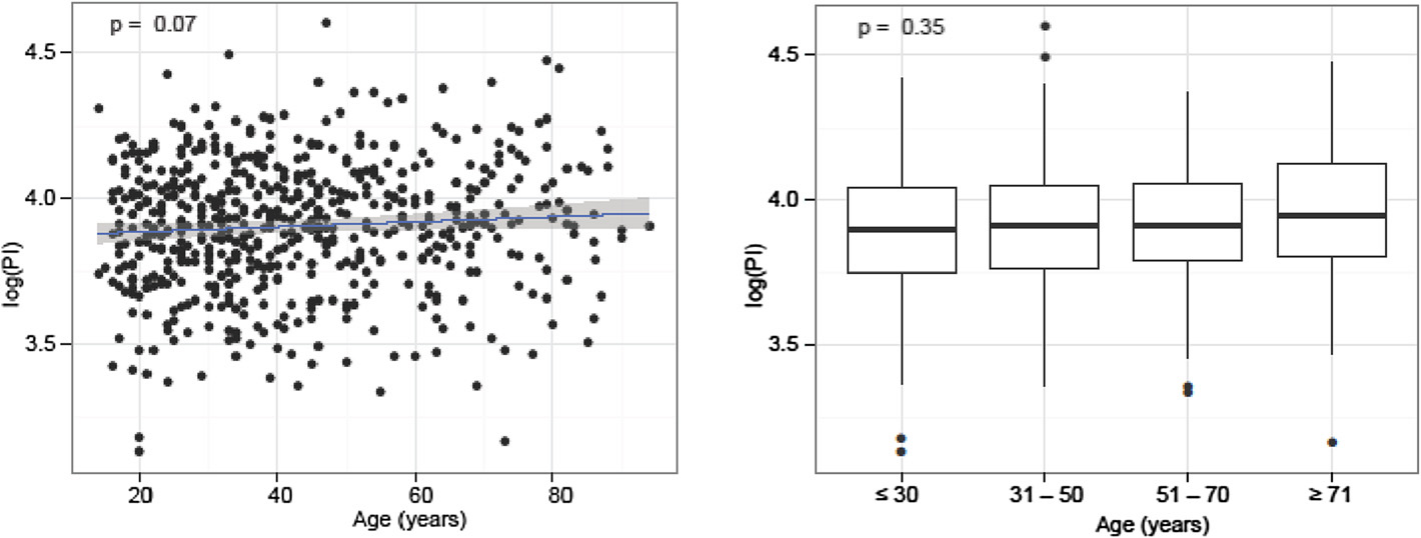
Pelvic Incidence (PI) and Age: There was no significant difference for PI and age.

There was no significant difference for PI and age (Figure 4). In the 314 individuals of the younger age group ≤ 40 years, the mean PI (50.1°) was slightly lower than the one (51.7°) in the 282 individuals of the older age group >40 years (p = 0.07) (Table 1). These two groups are relatively equally populated and statistical analysis may be assumed to have enough power. There was no significant difference (p = 0.35) for PI when grouping individuals into age groups of ≤ 30 years, 31–50 years, 51–70 years and ≥ 71 years either (Table 1).

3) PI and Gender

**Table 1:** There was no significant difference for PI and age (p = 0.07)

	**Overall**	**Females**	**Males**	**Mean PI**	**P Value**
≤ 40 years	314	103	211	50.1°	0.07
> 40 years	282	90	192	51.7°	
≤ 30 years	185	57	128	50.0°	0.35
31-50 years	226	70	156	50.6°	
51-70 years	124	40	84	51.3°	
≥ 71 years	61	26	35	53.3°	

In the 314 individuals of the younger age group ≤ 40 years, the mean PI (50.1°) was slightly lower than the one (51.7°) in the 282 individuals of the older age group ≥ 40 years.

**Figure 5:**
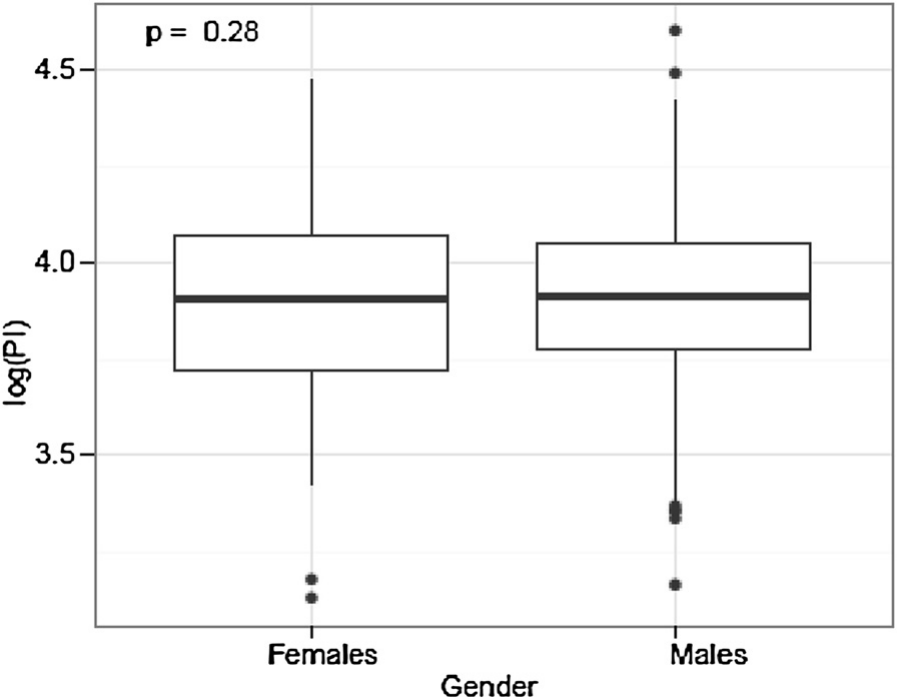
Pelvic Incidence (PI) and Gender: We did not find a significant difference for PI and gender.

We did not find a significant difference for PI and gender (p = 0.28) (Figure 5). The mean PI (50.3°) for 193 females was slightly lower than the one (51.1°) for 403 males (Table 2).

4) PI and LL

**Table 2:** We did not find a significant difference for PI and gender (p = 0.28)

	**Females**	**Males**	**P Value**
Overall	193	403	
Mean PI	50.3°	51.1°	0.28

The mean PI (50.3°) for 193 females was slightly lower than the one (51.1°) for 403 males.

**Figure 6:**
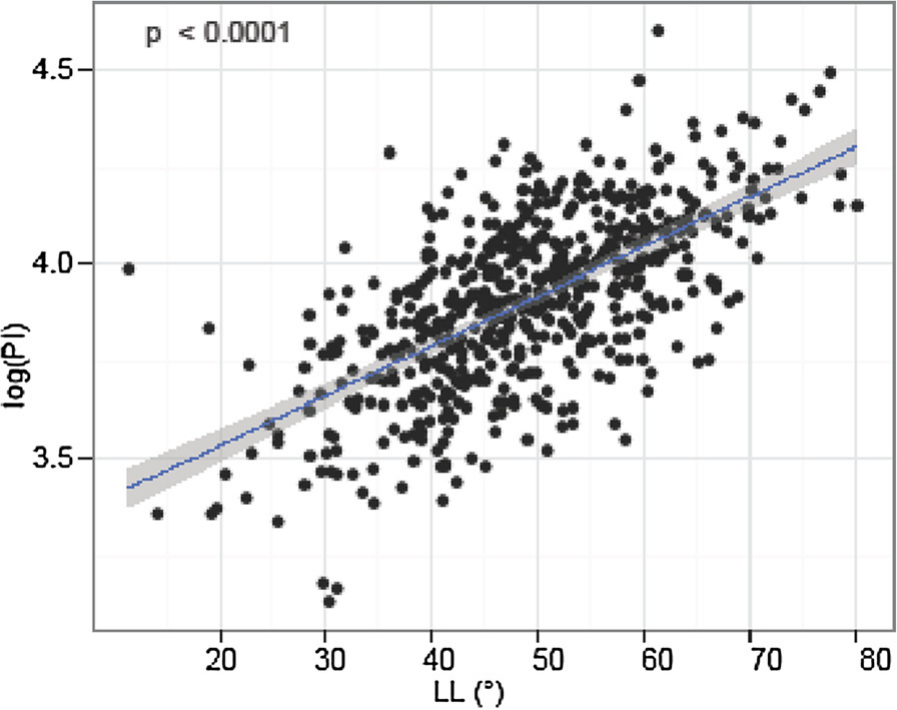
Pelvic Incidence (PI) and Lumbar Lordosis (LL): PI was significantly and linearly correlated with LL.

**Figure 7:**
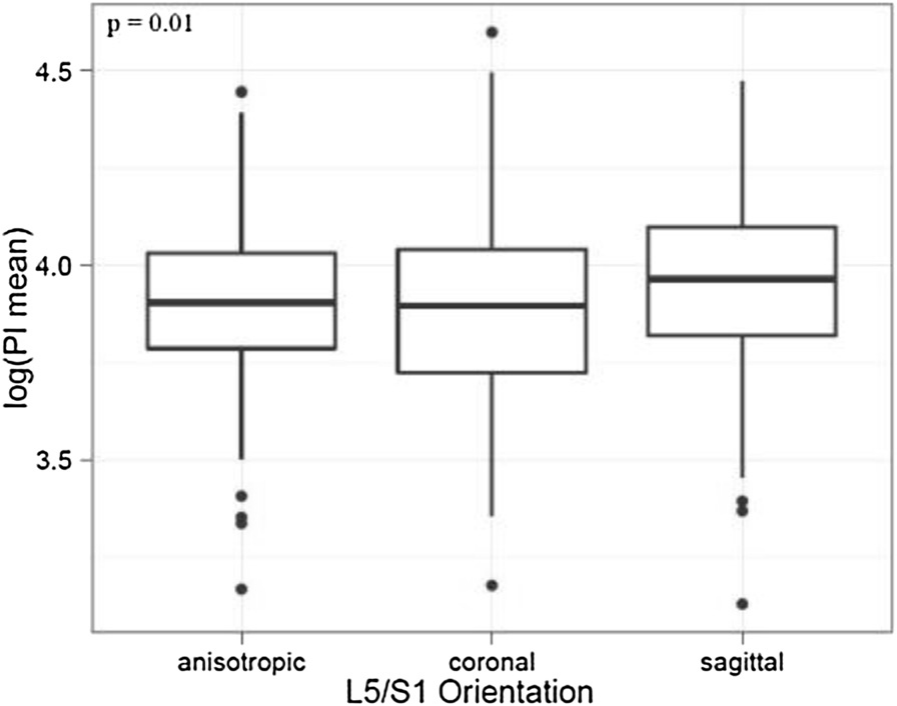
**Pelvic Incidence and FJ Orientation at L5/S1: There was a significant difference in the logarithm of the mean PI and FJ orientation at L5/S1.** The FJ orientation was labeled as coronal if angles were > 45° and sagittal if angles were ≤ 45°.

The mean value for LL was 48.9°. PI was strongly correlated with LL (p = < 0.0001, r = 0.625) (Figure 6 and Figure 7). The lower the PI, the less LL was present. The mean PI (45.5°) was lower for 307 individuals with a LL less than the mean value compared with the one (56.5°) for 287 individuals with a LL more than the mean value.

5) PI and FJ Arthritis and Orientation

**Figure 8:**
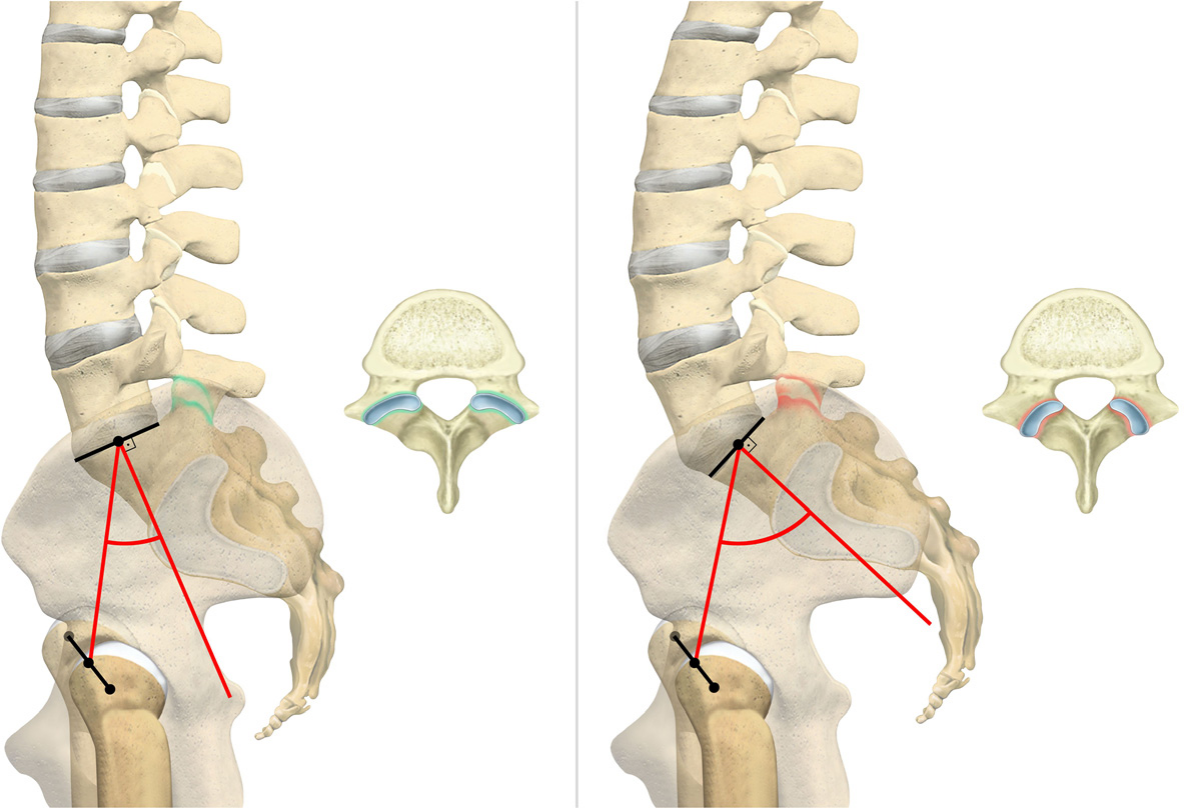
**Pelvic Incidence (PI), Facet Joint (FJ) Arthritis and Orientation at L5/S1: On the left side, low PI indicates a normal FJ and more coronal FJ orientation at the lower lumbar spine.** Contrarily, the right side shows increased PI with associated FJ arthritis and more sagittal FJ orientation at the lower lumbar spine.

**Figure 9:**
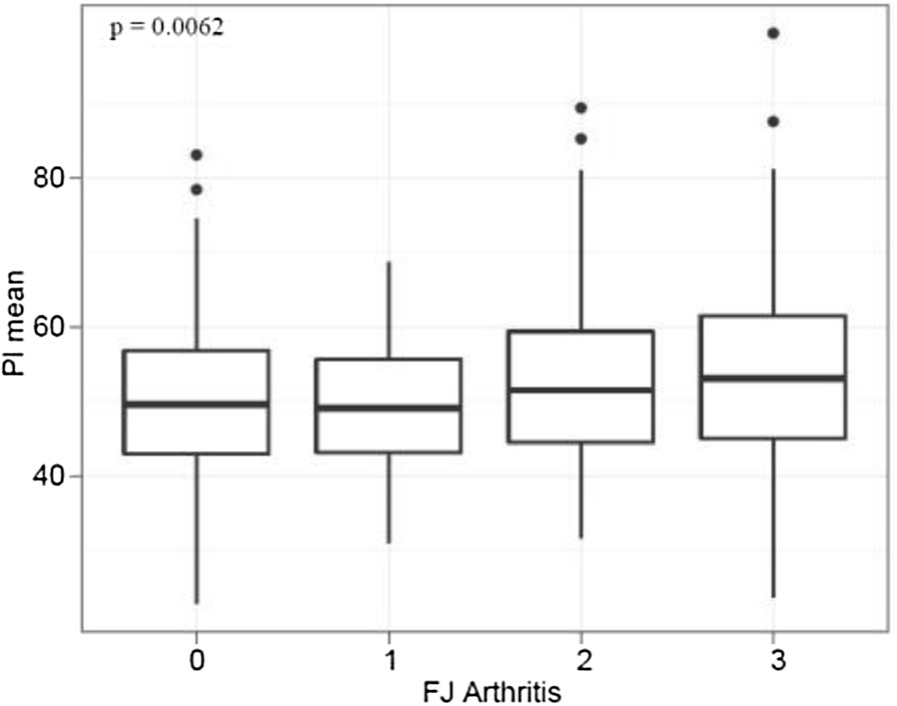
Pelvic Incidence (PI) and Facet Joint (FJ) Arthritis: PI and FJ arthritis displayed a significant linear correlation.

PI and FJ arthritis displayed a significant association (p = 0.0062, odds ratio 1.020 [95%-CI 1.005, 1.034]) (Figure 8 and Figure 7), whereby an increased PI was associated with increased FJ arthritis. Interestingly, the cut-off point ranged around a PI of 50°. The median PI of 49.6° (ICR 43°-56.8°) was lower in 293 individuals without FJ arthritis (grade 0) compared to the median PI of 50.4° (IQR 43.5°-59.3°) in 301 individuals with signs of FJ arthritis (grade 1–3). The unadjusted difference was 1.76° (95% CI: -0.02-3.5, p = 0.052). The median PI of 51.7° (IQR 43.2-57.0) was highest in 97 individuals with the most severe FJ arthritis (grade 3) compared to the median PI of 49.8° in individuals with a lower grade of FJ arthritis (grade 0–2). The unadjusted difference was 2.2 (95% CI: -0.18-4.59, p = 0.070). There was a significant difference in the logarithm of the mean PI and FJ orientation at the lower lumbar spine. Specifically, an increased PI was significantly associated with sagitally oriented FJs at L5/S1 (p = 0.01) (Figure 9 and Figure 7). However, comparison of the logarithm of the mean PI with FJ orientation at the upper lumbar spine did not reveal any significant differences (p = 0.71, 0.23 and 0.35 at L2/3, L3/4 and L4/5).

## Discussion

Our study investigated the largest sample of CT scans from different individuals in the literature in regard to PI and (1) its physiologic values, (2) age, (3) gender, (4) LL and marks the first study to investigate its relationship with (5) FJ arthritis and orientation. We were able to show that the (1) mean value for PI on CT scans ranges around 50.8°. PI was not significantly correlated with (2) age, nor (3) gender. However, we found a significant linear relationship between PI and (4) LL, (5) FJ arthritis and sagittal FJ orientation at the lower lumbar spine, namely L5/S1. PI and FJ orientation at the upper lumbar spine were not significantly correlated.

Limitations of our study attribute to the fact that all individuals presented to a trauma department. Even though a selection bias may be assumed, we did not include individuals with a fracture of the lumbar spine or pelvis. Furthermore, we did not pay special attention to degenerative disc disease since this has been investigated in previous studies [40,54,55]. Due to the retrospective nature of this study, we were not able to investigate which individuals showed clinical signs of FJ arthritis. Nevertheless, radiologic proof of FJ arthritis has not been clearly associated with back pain at all times [56-58]. A recent study by Vrtovec et al. [4] came to the conclusion that computerized measurements of PI in three dimensions are less variable than manual measurements. However, our measurements were carried out before publication of this study and we applied meticulous measurement techniques in order to achieve the most accurate values. We were not able to control for intra- and interobserver reliability, but measurements were carried out by two trained specialists in this field. Furthermore, the measuring technique is based on the same concept as previous studies [7,13], where the center of the superior end plate of S1 and the midpoint of the bicoxofemoral hip axis determine the PI. It should also be noted that our measurement technique did not require complex reconstruction of 3D images, but was based on sagittal CT slides of the pelvis. We did not specify the exact level or side of FJ arthritis since all levels and sides seemed to be affected in a similar fashion, with lower levels being slightly more frequently affected [59]. Even though our study included a similar number of individuals under and over 40 years, it comprised nearly twice as many males, which may be attributed to the fact that males are injured and present to a traumatology department more often [60]. However, we do believe that statistical conclusions can be drawn from this sample size. Furthermore, variable patient positioning in the CT scanner may lead to a misinterpretation of the exact middle of the spine and pelvis. We tried to solve this problem by choosing the same middle for the spine and sacrum. Anyhow, the same or at least very similar values can be calculated within several adjacent sagittal slides.

1) PI

Our meadian value for PI of 49.9° is in line with previous studies. A study by Peleg et al. [12,13] was the first to describe the PI in CT scans. They investigated 424 skeletons of articulated pelves as well as 20 individuals with CT scans and obtained mean values for PI of 52.8° and 57.1°. In a recent study by Vrtovec et al. [4], who successfullyevaluated CT images of 370 normal subjects, the mean value for PI was 47.1°. The large apan of values for PI indicated a relatively large natural variation. On the other hand, PI has been extensively studied on X-rays, even though overlap or magnification of structures my falsify the measured angle [7,17]. Vialle et al. [18] studied 300 lateral radiographs of volunteers and obtained a mean value for PI of 55°.

2) PI and Age

We did not find a significant difference for PI and age, even though younger (≤ 40 years) individuals had a slightly lower mean PI (50.1°) than older (≥ 40 years) individuals, who had a mean PI of 51.7°. Correspondingly, previous studies have shown that PI only increases until the age of ten and than stabilizes [19-21]. Mac-Thiong et al. [19] studied 180 healthy individuals between 4–18 years and found out that PI tends to increase until adolescence in order to keep an optimal sagittal balance and stabilizes into adulthood. Other reports mentioned an increase later on during life, especially when spondylolisthesis or sacral fractures are present [21-26]. Labelle et al. [23] studied 214 individuals with spondylolisthesis and found a linear relationship between PI and the severity of spondylolisthesis. In a study by Mendoza-Lattes et al. [25], 32 healthy teenagers were compared to 54 adults with spinal deformity and the PI was higher for the latter group. A previously mentioned study by Vrtovec et al. [4] found a linear increase of PI after skeletal maturity in normal subjects, suggesting a morphological change of the pelvis. However, our findings support the fact that PI does not change in adults as long as there is no evidence of deformity.

3) PI and Gender

Our study did not point out a significant gender diffence for PI, even though females (50.3°) showed slighthly lower values then males (51.1°). This is in line with previous studies [4,14,27,29,61]. In a large study, Mac-Thiong et al. [14] prospectively studied the spinal balance in 709 asymptomatic adults without spinal pathology using standing lateral radiographs, and found similar mean values for PI of 52.4° for females and 52.7° for males. Similarly, Janssen et al. [27] did not find a statistical gender difference, with mean values of 50° for 30 females and 53° for 30 males. On the other hand, Vialle et al. [18] reported a significant gender difference, whereby 110 females displayed a mean PI of 56° compared to 190 males with a mean PI of 53°. However, the difference of only 3° is far less than the commonly accepted measurement error of at least 5° [25]. Overall, we don’t believe that there is a significant gender difference in PI, because our study includes the largest sample size for the most accurate evaluation using CT scans, which is in line with the study with the largest last sample size for X-rays [14].

4) PI and LL

In our study, the mean value for LL was 48.9°. PI was significantly and linearly associated with LL (Figure 6 and Figure 7). Correspondingly, a predictive equation, LL = PI +9° (+/− 9°), has been recently suggested by Schwab et al. [30]. Aside from this linear correlation, our study suggests a lower value for LL than for PI, so we would advocate a modified equation, such as LL = PI – 2° (+/− 11°). This is in contrast to a study by Hanson et al. [24], who postulated that PI may increase to compensate for a gradual loss in LL with age. But, the increase in PI was seen by comparing 40 patients with spondylolisthesis to a control group of 20 adults and 20 adolescents. Thus, spinal deformity might have been a confounding factor.

5) Main hypothesis: PI and FJ Arthritis and Orientation

Our study marks the first study to investigate the association of PI with FJ arthritis and orientation. According to our hypothesis and as a novel finding, PI was significantly and linearly associated with FJ arthritis and sagittally oriented FJs at the lower lumbar spine, namely L5/S1 (Figures 7, 8 and 9). However, comparison of PI with FJ orientation at the upper lumbar spine did not reveal any significant differences. This is similar to a recent study by Toy et al. [37], who investigated 120 cadaver specimen and concluded that the highest quarter of pelvic lordosis is associated with FJ arthritis at L5/S1. According to Toy et al. [37], pelvic lordosis describes the angle between the pelvic radius line and a line tangent to the upper S1 endplate that intersected at the posterior superior corner of S1. However, they did not mention an association between PI and FJ arthritis. They also used a goniometer on the osteologic specimen, which may lead to more imprecise values. This is also in line with a study by Labelle et al. [23], who found a linear association between PI and spondylolisthesis. Our results support their hypothesis that an increased PI may lead to a higher mechanical stress on the FJs. An association of FJ arthritis with sagittal FJ orientation of the lower lumbar spine has been reported in a study of CT scans with 188 individuals by Kalichman et al. [34] and a similar study by Liu et al. [35] as well as a MRI study if 111 individuals by Fujiwara et al. [36]. Considering that the lowest three lumbar FJs carry the highest loads and LL leads to higher contact force on the FJs [32], it may be postulated that increased PI may also lead to higher contact force on the lower FJs and cause FJ arthritis along with more sagittal FJ orientation. Individuals with increased PI may therefore be at high risk for FJ arthritis at the lower lumbar spine. While FJ arthritis may be considered a degenerative disease, more sagittal FJ orientation of the lower lumbar spine may be a balancing mechanism.

The establishment of a neutral upright sagittal alignment with the pelvis and spine in sync is essential in the management of spinal disorders [5,6]. Our study aids in the ongoing process [16] of defining the optimal spinal balance. It validates that PI remains a key parameter in sagittal balance and provides another mean value in a large patient population. We also present an easy method for quick and accurate evaluation of PI on sagittal slices of CT scans that does not require complicated reconstruction of 3D images. Patients with increased PI are more likely to present with FJ arthritis and possibly from associated back pain. Once these patients with increased PI (and LL) become symptomatic, orthopaedic (trauma) surgeons may consider FJ infiltration and/or establishing less lordosis with percutaneous instrumentation, where available, in order to restorce spino-pelvic balance and prevent FJ arthritis if they feel that this may cause problems for the patient. In these trauma patients with increased PI (and LL), a fracture at the lumbar spine in need of spinal surgery, spondylodesis may be preferred over percutaneous instrumentation because these patients are more likely to suffer from FJ arthritis and its related pain.

## Conclusion

In conclusion, our study showed that the mean value for PI on CT scans ranges around 50.8°. PI is neither significantly correlated with age nor gender. However, this is the first report showing that PI is significantly and linearly associated with LL, FJ arthritis and sagittal FJ orientation at the lower lumbar spine. Increased PI may lead to higher contact force on the lower lumbar FJs and cause FJ arthritis along with more sagittal FJ orientation. Individuals with increased PI and (and increased LL) may therefore be at high risk for FJ arthritis at the lower lumbar spine. Patients with increased PI (and increased LL) could benefit from corrective surgery and spondylodesis, once symptomatic or in the event of trauma.

## Abbreviations

PI: Pelvic incidence; FJ: Facet joint; LL: Lumbar Lordosis.

## Competing interests

The authors declare that they have no competing interests.

## Authors’ contributions

TJ: Conception and design, acquisition of data, analysis and interpretation of data, drafting the manuscript, revising the manuscript, final approval of the version to be published. JG: Acquisition of data. SB: Conception and design. KS: Revising the manuscript, statistics. DLN: Revising the manuscript. CMLW: Conception and design, analysis and interpretation of data, revising the manuscript, final approval of the version to be published. All authors read and approved the final manuscript.

## Pre-publication history

The pre-publication history for this paper can be accessed here:

http://www.biomedcentral.com/1471-2342/13/34/prepub
